# Thermally-triggered Dual *In-situ* Self-healing Metallic Materials

**DOI:** 10.1038/s41598-018-19936-4

**Published:** 2018-02-01

**Authors:** JeongTae Kim, Hee Jin Kim, Sung Hwan Hong, Hae Jin Park, Young Seok Kim, Yun Jung Hwang, Yeon Beom Jeong, Jun-Young Park, Jin Man Park, Baran Sarac, Wei-Min Wang, Jürgen Eckert, Ki Buem Kim

**Affiliations:** 1 Department of Nanotechnology and Advanced Materials Engineering, Sejong University, 209, Neungdong-ro, Gwangjin-gu, Seoul, 05006 Republic of Korea; 2 Erich Schmid Institute of Materials Science, Austrian Academy of Sciences, Jahnstraße 12, A-8700 Leoben, Austria; 3 Department Materials Physics, Montanuniversität Leoben, Jahnstraße 12, A-8700 Leoben, Austria; 4 Global Technology Center, Samsung Electronics Co., Ltd., 129, Samsung-ro, Yeongtong-gu, Suwon-si, Gyeonggi-do 443-742 Republic of Korea; 5 Key Laboratory for Liquid-Solid Structural Evolution and Processing of Materials, Ministry of Education, Shandong University, 17923 Jingshi Road, Jinan, 250061 China

## Abstract

The microstructural evolution and crack filling phenomena of (Al_81_Cu_13_Si_6_)_100−*x*_(Sn_57_Bi_43_)_*x*_ (*x* = 0, 1, and 3 at.%) composites was investigated. The Sn and Bi elements were selected by considering the ability for liquid phase separation when combined with Al, Cu, and Si. Because of liquid phase separation, both Al-Cu-Si-rich L_1_ and Sn-Bi-rich L_2_ phases separately solidified at different temperatures yielding a trimodal eutectic structure in the cast alloys. The Sn and Bi elements have high mobilities due to the large interface of the eutectic microstructure and tend to strongly diffuse towards higher strained region during heat treatment. Furthermore, the mobile Sn and Bi elements in the Al-Cu-Si-based bimodal eutectic structure evidently fill cracks during warm rolling at 423 K. These results reveal that the developed alloy system has simultaneously dual self-healing characteristics, derived from the both precipitated Sn-Bi-rich particles and low melting agent, and the proposed alloy design based on liquid phase separation provides a novel strategy for creating self-crack filling metallic materials.

## Introduction

Ultrafine eutectic composites have attracted considerable attention due to their high strength, good thermal properties and favorable wear resistance compared with coarse-structured materials^1–5^. Especially, their higher volume fraction of interfaces is one of the reasons for the novel properties. However, these materials generally suffer from insufficient plasticity and reduced toughness during deformation, which hampers their widespread application and makes it nearly impossible to subject them to conventional secondary treatments such as rolling or forming operations due to pronounced crack generation^6,7^. In order to improve their deformability, many studies have been performed to improve the plasticity by optimizing the microstructural evolution. For instance, ultrafine eutectic composites with micro-scale primary dendrites have been reported^1,8–11^. Because the micro-scale dendrites act as toughening phase, they can release the applied stress at the early deformation stage and generate multiple shear bands as well as impede the propagation of shear bands. Moreover, the volume fraction of primary dendrites can be controlled by simple alloy design, which means that the mechanical properties are controllable even though the enhancement of the plasticity often inevitably leads to decreased strength. Furthermore, bimodal eutectic structures with structural and/or chemical heterogeneities show enhanced plastic deformability without significant deterioration of strength^12–17^. These findings indicate that the mechanical properties can be tailored via the control of microstructural heterogeneities (structural and chemical), since the length-scale, volume fraction and morphology of the eutectic structure has a strong impact on the mechanical properties^12–17^. However, so far, as mentioned before, there have been no attempts to form such eutectic alloys via conventional rolling processes due to their fragile nature induced by crack activation during forming, which renders rolling processes nearly impossible.

Recently, self-healing materials, i.e. materials which have the ability to heal or repair spontaneously surface or internal damage, have been highlighted as smart materials^18–20^. The concepts of self-healing can be divided into two types: autonomous and non-autonomous self-healing. Autonomously self-healing materials, which are almost completely polymeric materials, can heal or repair themselves when they have damage at the surface or in their interior^18,19^. This process does not require any additional external stimulus. In contrast, non-autonomous self-healing materials require external triggers such as heat or light. Especially non-autonomous self-healing in metallic materials, such as the Fe-Au alloy, MAX phase, Nanoreservoirse, Sn-based composite with NiTi shape memory wires and so on, has been investigated with concepts following three basic ideas: (i) precipitation from supersaturated solid solutions to fill cracks and voids, (ii) introduction of shape memory alloy wires as reinforcements in the metallic matrix, and (iii) using low melting temperature metals as curing agent of the metallic matrix^20–29^.

On the basis of these considerations, ultrafine eutectic composites with homogeneously dispersed liquid phase separated dispersions were designed. The designed novel alloys are Al-Cu-Si-Sn-Bi systems with two different melting temperatures. These alloys can be expected to act as self-healing materials, which are reinforced by phases of both low melting temperature metals (Sn and Bi) via precipitation from supersaturated solid solutions. The eutectic alloys contain a much higher volume fraction of interfaces than conventional coarse-grained materials, suggesting a considerable amount of rapid diffusion paths^30^. Therefore, the solute atoms in the supersaturated solid solution have a higher mobility through the interface of the eutectic structure to high strain regions^21^.

The Al-Cu-Si-Sn-Bi eutectic system forms liquid separated Sn-Bi eutectic phases in the Al-Cu-Si eutectic matrix^31–34^. These Sn-Bi eutectic phases have low melting temperatures, in a temperature range of 411.15 to 412 K^35^. Hence, Al-Cu-Si-Sn-Bi eutectic alloys exists in liquid + solid state at 423 K and the low melting Sn-Bi eutectic phases can act as healing agent in a respectively high-melting temperature alloy matrix composite at 423 K^21^.

In this study (Al_81_Cu_13_Si_6_)_100−*x*_(Sn_57_Bi_43_)_*x*_ alloys with *x* = 0, 1, and 3 at.% are investigated with the aim to ultrafine multi-modal eutectic composites and to understand their microstructural evolution and self-healing properties. In order to control the microstructure, the instability of solidification caused by solute partitioning and liquid separation is considered. In general, Al-Cu-Si composites are known as one type of ultrafine bimodal eutectic composites composed of two kinds of eutectic structures with different constituent phases (Al + Al_2_Cu and Al + Al_2_Cu + Si) and length-scales^12,36–38^. The Sn and Bi elements are selected due to their liquid phase separation relationship with Al, Cu, and Si, where both L_1_ (Al, Cu, and Si-rich liquid) and L_2_ (Sn and Bi-rich liquid) phases can coexist in the liquid state. Their melting and solidification behavior is distinctly different and completely separated^31,32^. Following these considerations, the aim of the present investigation is to analyze the microstructural evolution of multi-modal Al-Cu-Si-Sn-Bi alloys and to evaluate their ability for crack filling during heat treatment and warm rolling at 423 K.

## Results

**Figure 1:**
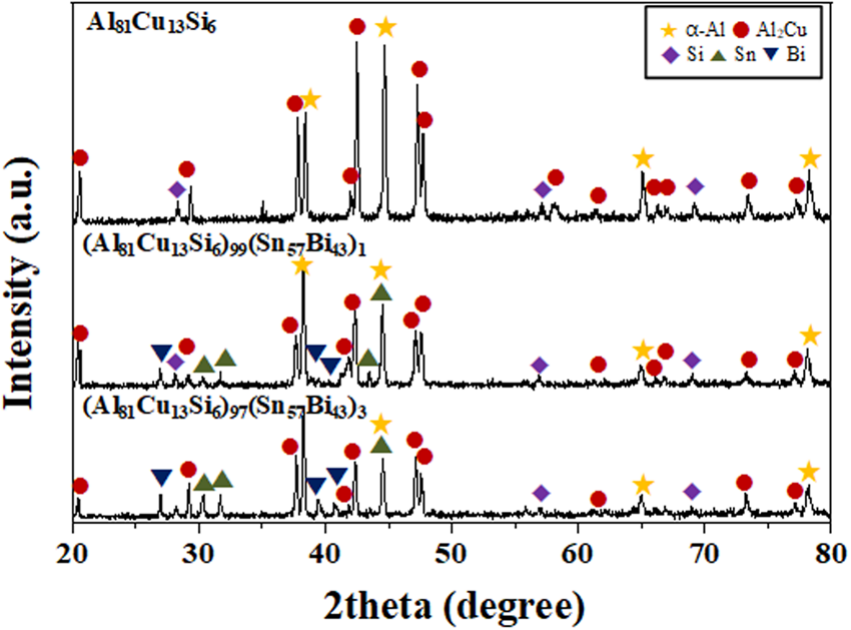
X-ray patterns obtained for as-cast (Al_81_Cu_13_Si_6_)_100−*x*_(Sn_57_Bi_43_)_*x*_ (*x* = 0, 1, and 3 at.%) composites; The ternary Al-Cu-Si alloy displays peaks of α-Al, Al_2_Cu, and Si phase. The Sn-Bi-containing alloys exhibit additional peaks corresponding to Sn and Bi phases besides with the reflections of α-Al, Al_2_Cu, and Si.

**Figure 2:**
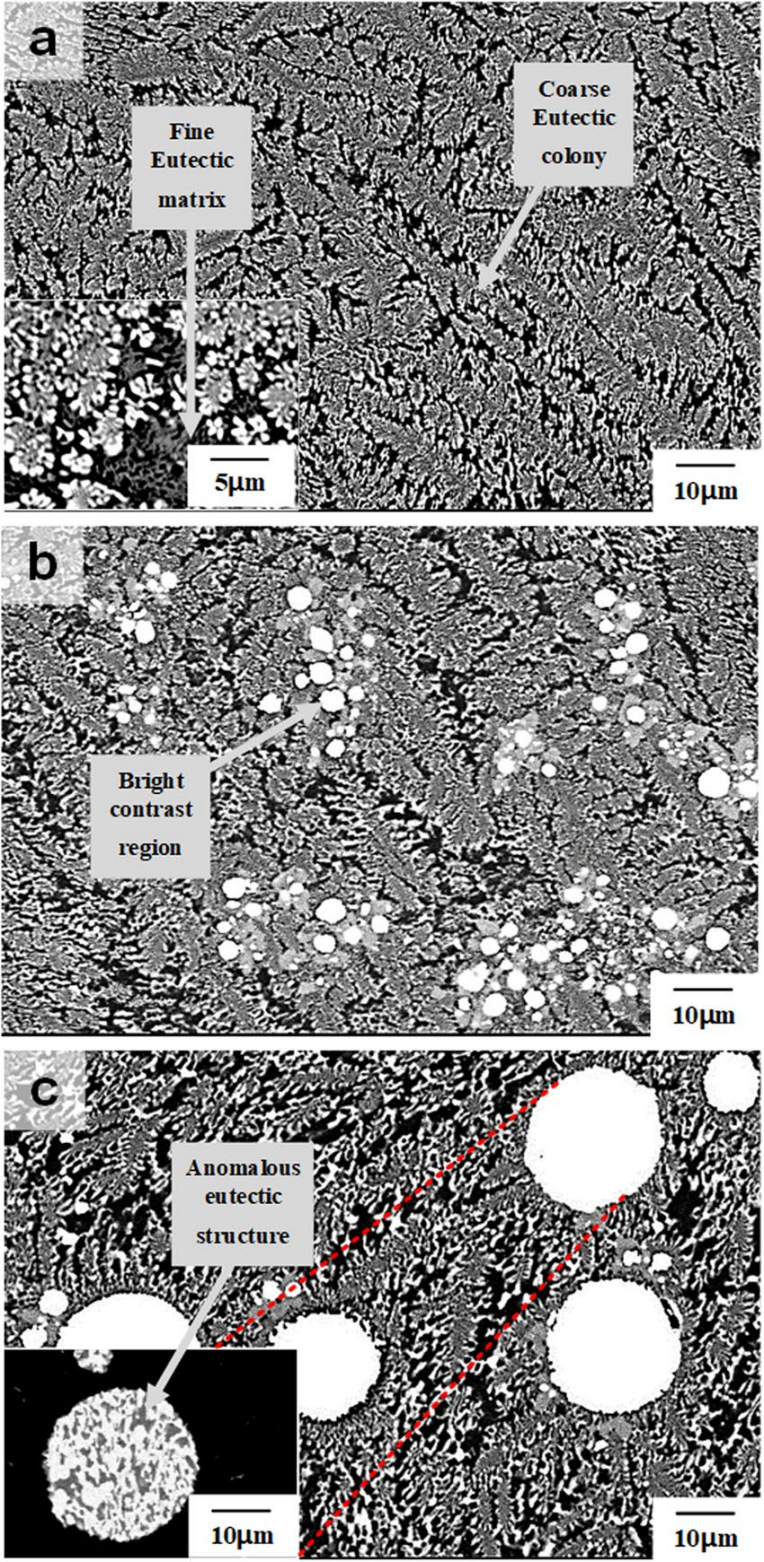
Microstructure for as-cast (Al_81_Cu_13_Si_6_)_100−*x*_(Sn_57_Bi_43_)_*x*_ (*x* = 0, 1, and 3 at.%) composites; (**a**) The SEM image of the Al_81_Cu_13_Si_6_ alloy displays a bimodal eutectic structure with different length-scale and constituent phases. (**b**) The SEM image of the (Al_81_Cu_13_Si_6_)_99_(Sn_57_Bi_43_)_1_ alloy exhibits brighter contrast particles, which are Sn-Bi-rich phases, in the bimodal eutectic matrix. (**c**) The SEM image of the (Al_81_Cu_13_Si_6_)_97_(Sn_57_Bi_43_)_3_ alloy also displays a trimodal eutectic microstructure (bimodal Al-Cu-Si + anomalous Sn-Bi) with increased volume fraction and size of brighter contrast areas.

**Figure 3:**
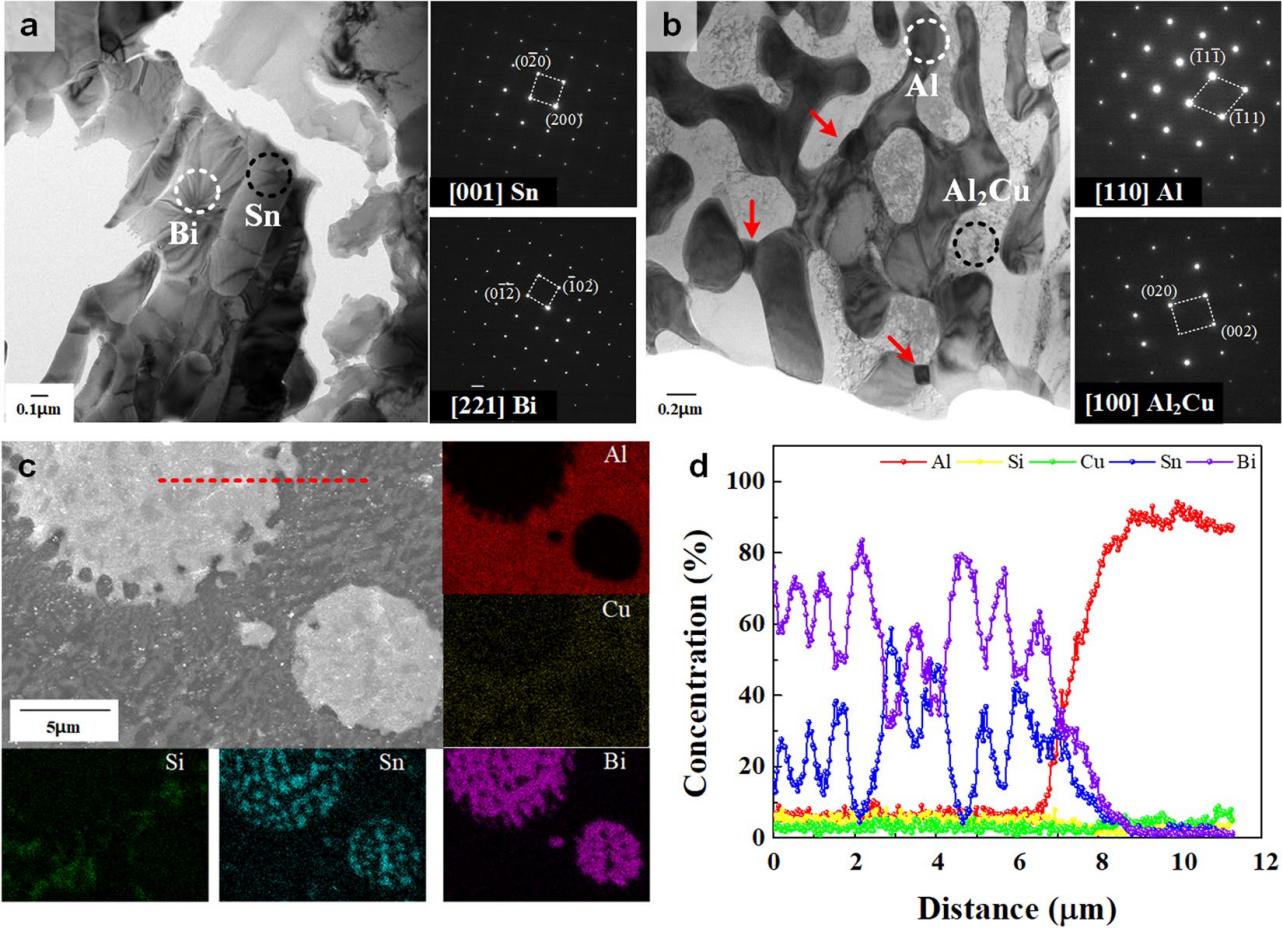
TEM and EDX results for as-cast (Al_81_Cu_13_Si_6_)_100−*x*_(Sn_57_Bi_43_)_*x*_ (*x* = 0, 1, and 3 at.%) composites; (**a**) TEM results showing that the brighter contrast droplets consist of Sn and Bi phases. (**b**) Small Sn and/or Bi particles are located in the Al-Cu-Si rich bimodal eutectic matrix originated from the monotectic reaction. (**c**) EDX mapping and (**d**) line scan results of the different Al, Cu, Si, Sn, and Bi elements on the (Al_81_Cu_13_Si_6_)_97_(Sn_57_Bi_43_)_3_ alloy.

Figure 1 shows the XRD patterns of the as-cast (Al_81_Cu_13_Si_6_)_100−*x*_(Sn_57_Bi_43_)_*x*_ composites with *x* = 0, 1, and 3 at.%. The diffraction peaks of the Al_81_Cu_13_Si_6_ ultrafine bimodal eutectic composite are identified as a mixture of a face-centered cubic (f.c.c.) ${\rm{\alpha }}$-Al solid solution (*Fm* $\bar{3}$ *m*, a = 0.4039 nm), a body-centered tetragonal (b.c.t.) ${\rm{\theta }}$ phase (Al_2_Cu) (*I4/mcm*, a = 0.6064 nm and c = 0.4873 nm), and a diamond cubic (d.c.) Si phase (*Fd* $\bar{3}$ *m*, a = 0.543 nm), similar to previous investigations^12,36–38^. In contrast, in case of (Al_81_Cu_13_Si_6_)_99_(Sn_57_Bi_43_)_1_ and (Al_81_Cu_13_Si_6_)_97_(Sn_57_Bi_43_)_3_ alloys, additional diffraction peaks corresponding to a tetragonal Sn phase (*I41/amd*, a = 0.5831 nm, c = 0.3182 nm) and a rhombohedral Bi phase (*R* $\bar{3}$ *m*, a = 0.4535 nm and c = 1.1814 nm) are observed besides ${\rm{\alpha }}$-Al, ${\rm{\theta }}$ (Al_2_Cu), and Si phases^31,33,34^. No diffraction peaks of intermetallic compounds containing Sn or Bi are observed. The SEM image displayed in Fig. 2a shows that the Al_81_Cu_13_Si_6_ composite has a typical bimodal ultrafine eutectic structure as it was also found in our previous research^12,36–38^. The bimodal eutectic microstructure, which is corresponding to the different constituent phases and a length-scale heterogeneity of the lamellar spacing, have coexisted as shown the inset in Fig. 2a. This alloy has the 2 difference eutectic structures such as the coarse eutectic colony composed of α-Al + Al_2_Cu and fine eutectic matrix which has the α-Al + Al_2_Cu + Si as a constitution^12,36–38^. On the other hand, the microstructure of the Sn and Bi added alloys in the Al_81_Cu_13_Si_6_ ultrafine bimodal eutectic composites exhibits the formation of white spherical phases with different sizes and volume fraction, as shown in Fig. 2b and c. Moreover, the volume fraction of the brighter spherical phases as determined by image analysis increases from 0.6 to 13 vol% when the Sn and Bi contents increase from 1 to 3 at.%. Moreover, the brighter contrast regions (spherical phases) display anomalous eutectic microstructures, as shown in the inset in Fig. 2c. These findings indicate that the Sn-Bi-containing alloys have a multi-modal eutectic microstructure comprised of coarse eutectic colonies, fine eutectic matrix, and spherical anomalous eutectic droplets. To further identify the microstructural features of the Al-Cu-Si-Sn-Bi ultrafine multi-modal eutectic composites, TEM was performed. Figure 3a,b present bright field (BF) images and SAED patterns for the (Al_81_Cu_13_Si_6_)_97_(Sn_57_Bi_43_)_3_ composite. The BF image obtained from the spherical anomalous eutectic droplets clearly shows the typical anomalous eutectic structure, which coincides with the SEM images (Fig. 2c). The SAED patterns in Fig. 2a correspond to the [001] zone axis of the tetragonal structure of the Sn phase and the [2 $\bar{2}$ 1] zone axis of the rhombohedral structure of the Bi phase, respectively. Moreover, the EDX results reveal that the average compositions of the Sn and Bi phases in the spherical anomalous eutectic droplets are Sn_86.3_Bi_9.3_Al_3.9_Cu_0.3_Si_0.2_ and Bi_98.4_Sn_0.6_Al_0.1_Cu_0.2_Si_0.7_, respectively. This indicates that the spherical anomalous eutectic droplets consist of eutectic Sn and Bi phases, similar as it is frequently observed for Sn-Bi solders^39^. Moreover, some small particulate phases are distributed among the inter-lamellar areas of the Al-Cu-Si-rich eutectic structure composed of the [110] zone axis of the Al phase with bright contrast and the [100] zone axis of the Al_2_Cu phase with dark contrast, as shown in Fig. 3b by the red line arrows. The different contrast in the eutectic α-Al phases is the quenched-in defect which is formed during casting process using water-cooled copper mold. These small particles were identified to be enriched in Sn by EDX (Sn_83.9_Al_11.5_Cu_4.6_). Most likely, these Sn-rich small particles are formed by precipitation during the monotectic reaction^31,33,34^. Figure 3c,d show the EDX mapping of the each elements and line scanning results. The EDX mapping results clearly verify a high concentration of Sn and Bi with the spherical droplet, and beside the relatively small amount of Sn element is observed in the matrix area. These results indicate that the Sn elements are present in the matrix as a solute. The line scanning results obtained from the red dashed line in Fig. 3c demonstrate the variation of element concentration near the interface between the Sn-Bi-rich droplet and Al-rich matrix. The pronounced compositional difference between the droplet and matrix, as well as the concentration fluctuations indicating the alternating eutectic structure were clearly came out. The detailed EDX results are shown in Supplementary Fig. S1 and S2.

**Figure 4:**
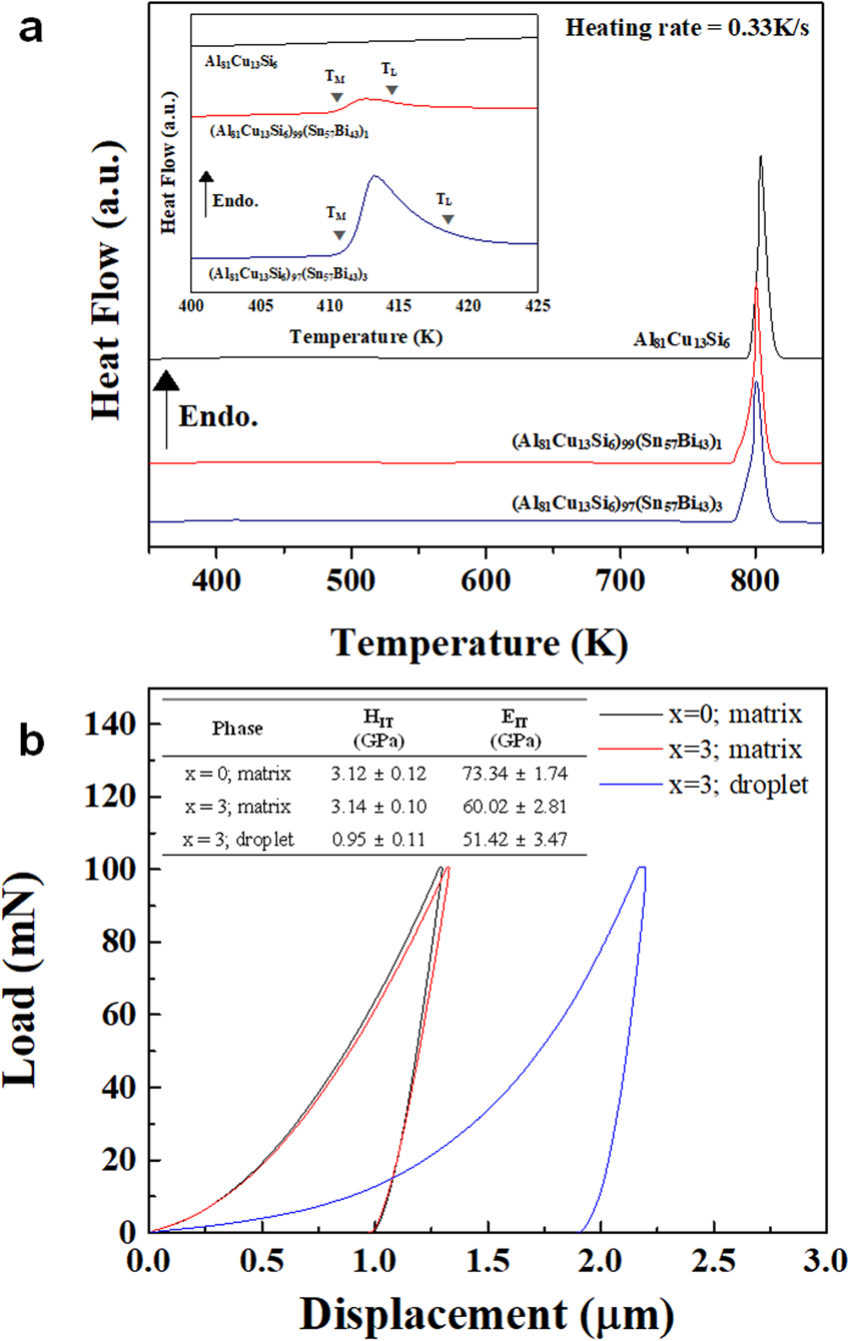
(**a**) DSC traces showing 2 different thermal events upon the heating, indicating liquid phase separation between (Al-Cu-Si) and (Sn-Bi). (**b**) Typical load-displacement (*P-h*) curves obtained from the matrix and droplet areas of the Al_81_Cu_13_Si_6_ and (Al_81_Cu_13_Si_6_)_97_(Sn_57_Bi_43_)_3_ alloys.

In order to investigate the melting and solidification behavior, DSC analysis of the as-cast (Al_81_Cu_13_Si_6_)_100−*x*_(Sn_57_Bi_43_)_*x*_ composites with *x* = 0, 1, and 3 at.% at a heating rate of 0.33 K/s was performed (as shown in Fig. 4a). For the Al_81_Cu_13_Si_6_ ultrafine bimodal eutectic composite, the DSC curve exhibits one endothermic peak around 790 K, associated with the melting temperature of the Al_81_Cu_13_Si_6_ ultrafine bimodal eutectic composition^40^. On the other hand, the Sn-Bi-containing (Al_81_Cu_13_Si_6_)_99_(Sn_57_Bi_43_)_1_ and (Al_81_Cu_13_Si_6_)_97_(Sn_57_Bi_43_)_3_ ultrafine multi-modal eutectic composites display two different endothermic peaks. Upon heating a first endothermic peak appears in the DSC trace at around 410 K (cf. the magnified inset in Fig. 4a). This event is correlated with melting of the Sn-Bi anomalous eutectic phases, as verified by the comparison with the Sn-Bi phase diagram^35^. The second event observed at around 790 K corresponds to the melting of the Al-Cu-Si bimodal eutectic area. These findings reveal that the melting and solidification behavior of the Sn-Bi-containing alloys are absolutely separated which originates from liquid phase separation between Al-Cu-Si and Sn-Bi^31–34^. To ascertain the influence of additional Sn and Bi on the mechanical properties, the nanoindentation was performed. Figure 4b displays the load-displacement curves obtained from the matrix areas of the Al_81_Cu_13_Si_6_ and (Al_81_Cu_13_Si_6_)_97_(Sn_57_Bi_43_)_3_ alloys and the droplets of the (Al_81_Cu_13_Si_6_)_97_(Sn_57_Bi_43_)_3_ alloy. The measured indentation hardness and modulus values of the Al_81_Cu_13_Si_6_ alloy were 3.12 ± 0.12 GPa, 73.34 ± 1.74 GPa, respectively. The values obtained from the matrix area of (Al_81_Cu_13_Si_6_)_97_(Sn_57_Bi_43_)_3_ alloy were 3.14 ± 0.10 GPa of indentation hardness and 60.02 ± 2.81 GPa of indentation modulus and the values of the droplet exhibit the much smaller indentation hardness and modulus of 0.96 ± 0.11 GPa and 51.24 ± 3.47 GPa, respectively. The detailed nanoindentation properties are summarized in Supplementary Fig. S3 and Table S1.

**Figure 5:**
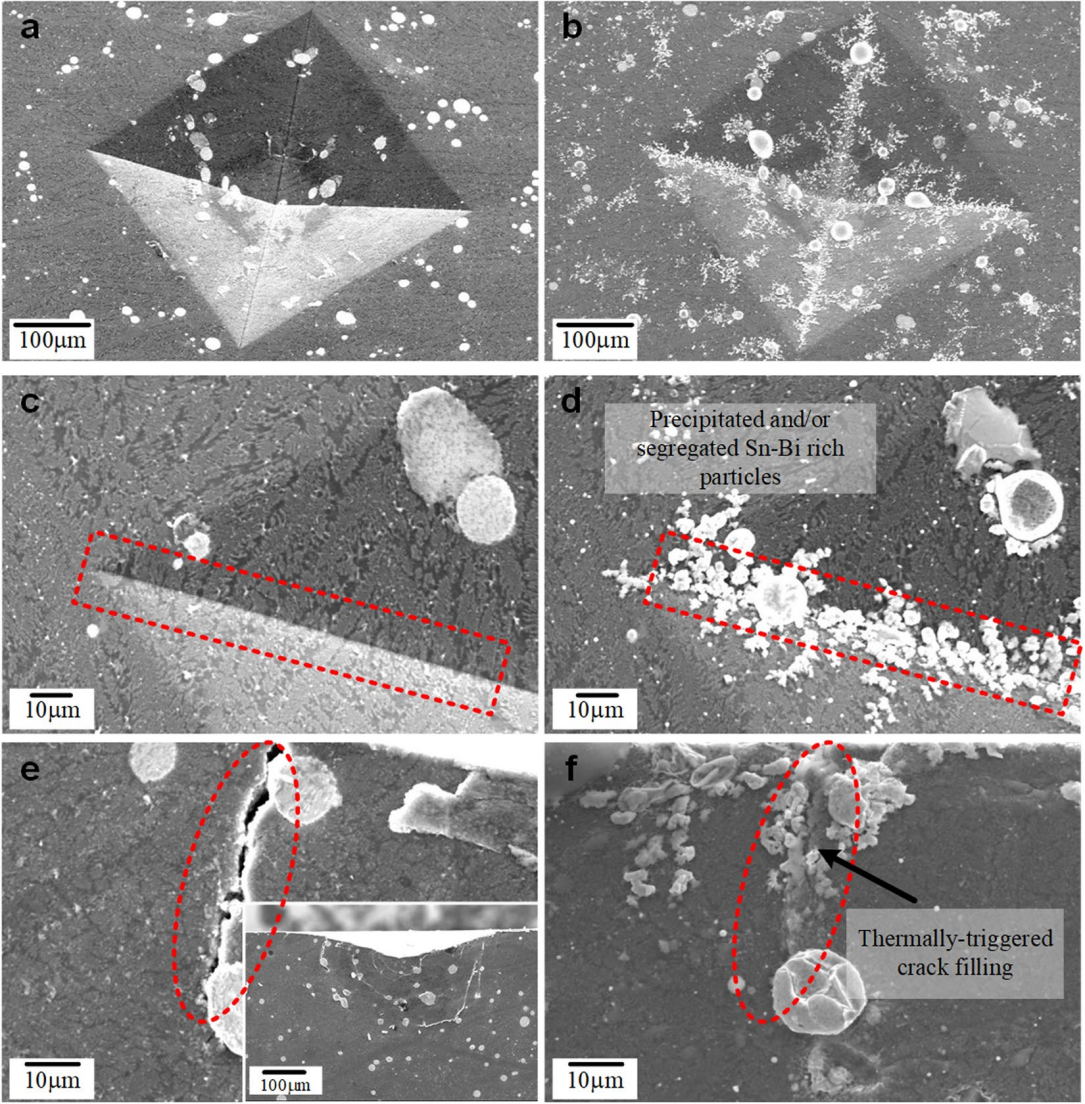
SEM images of hardness indents and subsurface deformation underneath the indents obtained for as-deformed and heat treated (Al_81_Cu_13_Si_6_)_97_(Sn_57_Bi_43_)_3_ samples; (**a**,**b**) SEM micrographs of the as-deformed zone. (**b**,**d**) After heat treatment at 423 K for 25 min segregated Sn-Bi-rich particles are found along the corner of the diamond indenter, which indicates the tendency to form Sn and Bi-rich precipitates around high stress/strain regions. (**e**,**f**) The cracks generated underneath the indents are filled by Sn-Bi-rich phases during heat treatment at 423 K for 25 min.

Figure 5 displays SEM images of the deformation zones on the top surface (a, c) and underneath the indent (e) after Vickers indentation at 0.49 kN load. The SEM images in Fig. 5b,d and f were obtained from the same location of specimens that were heat treated at 423 K for 25 min. As shown in Fig. 5a and b, particles have formed on the surface areas after heat treatment in the vacuum furnace. These particles are mainly found near the corner of the indents, i.e. in severely deformed areas, as marked by the red dotted square in Fig. 5c and d. We believed that these particles have diffused out of the liquid phase separated Sn-Bi eutectic region and/or the supersaturated Al-Cu-Si rich bimodal eutectic matrix, which indicates that the Sn-Bi anomalous eutectic region and/or Sn-Bi-rich areas can move to high strain regions such as voids, near the cracks, and other open-volume defects during the heat treatment. Furthermore, the micron-scale cracks underneath the Vickers indents were filled after the heat treatment at 423 K during 25 min, as shown in Fig. 5e and f. These observations indicate that the open-volume defects generated by deformation can be filled or recovered by thermally-triggered diffusion and/or precipitation processes during heat treatment at 423 K.

Another process that can be suggested is that the Sn-Bi anomalous eutectic regions may act as a healing agent due to their low melting temperature. From the DSC curves (Fig. 4a) it is clear that the Sn-Bi anomalous eutectic regions are completely melted at 423 K, which indicates that the Sn-Bi-containing alloys should be in Sn-Bi-rich liquid + solid (Al-Cu-Si bimodal eutectic) state at 423 K. Altogether, these results indicate that (Al_81_Cu_13_Si_6_)_100−*x*_(Sn_57_Bi_43_)_*x*_ (*x* = 1 and 3 at.%) self-healing materials containing a healing agent with low melting point can be synthesized *in-situ* by a simple casting process without any *ex-situ* method such as creating holes or using hollow micro-tubes to supply the healing agent ‒ some of the approaches that have been reported so far^19–21^.

**Figure 6:**
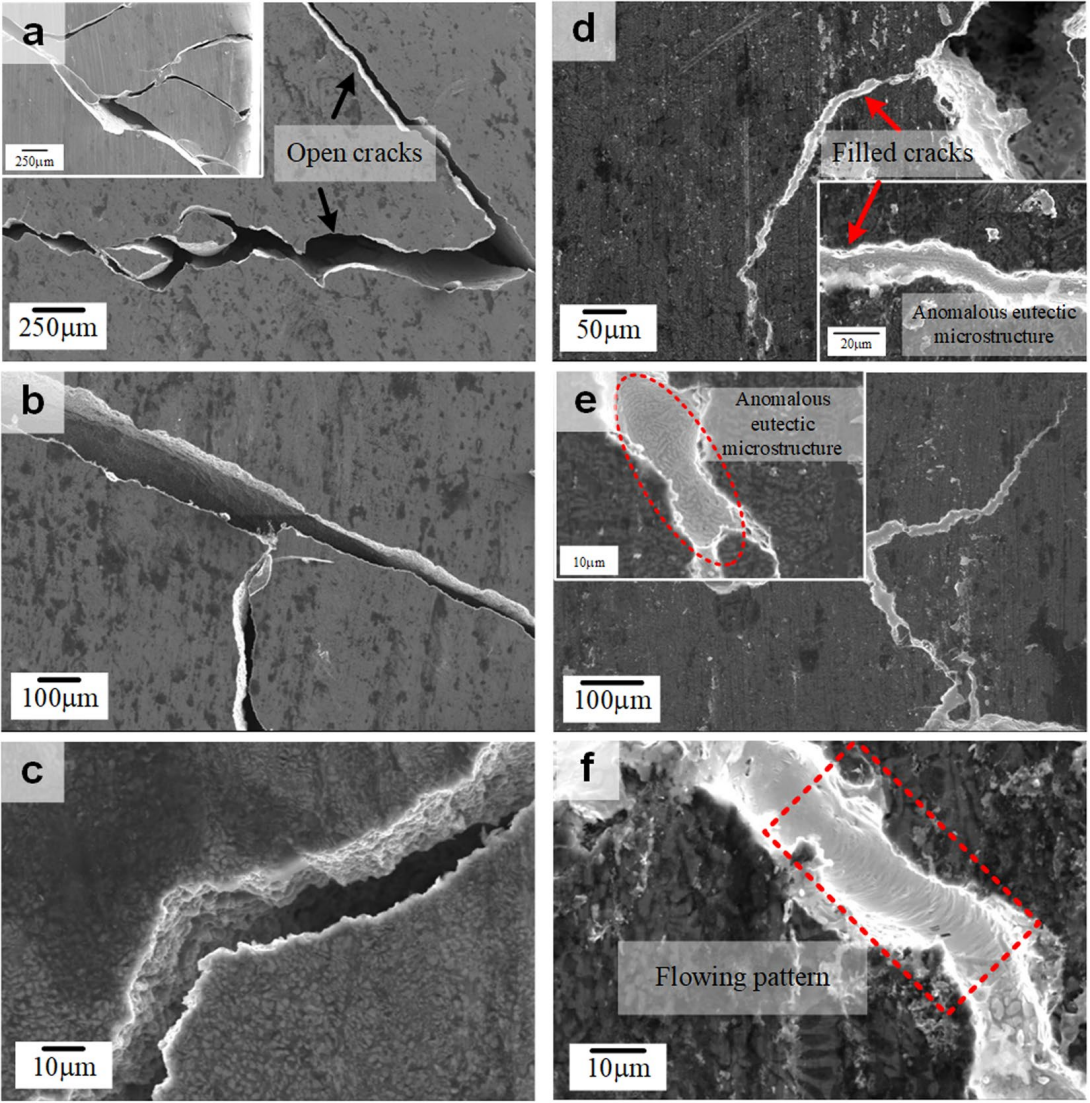
SEM images after warm rolling at 423 K with 10% reduction ratio for (**a**–**c**) Al_81_Cu_13_Si_6_ and (**d**–**f**) (Al_81_Cu_13_Si_6_)_97_(Sn_57_Bi_43_)_3_ alloys; (**a**–**c**) Micro-cracks have occurred during warm rolling in case of the Al_81_Cu_13_Si_6_ alloy and remain open cracks. (**d**–**f**) Micro-cracks formed during warm rolling are filled by the anomalous eutectic structure, indicating dynamic crack filling by the *in-situ* low melting temperature healing agents, which contain Sn and Bi.

To demonstrate dynamic crack filling capabilities, warm rolling was performed at 423 K with 10% reduction ratio. After the warm rolling process SEM analysis was performed on deformed Al_81_Cu_13_Si_6_ and (Al_81_Cu_13_Si_6_)_97_(Sn_57_Bi_43_)_3_ samples. Figure 6a–c display SEM images obtained for the as-rolled Al-Cu-Si bimodal eutectic alloy, showing numerous micron-scale cracks formed at the first rolling step. This indicates that even though the Al-Cu-Si bimodal eutectic alloy shows enhanced plastic deformability as frequently observed eutectic alloys^12,36–38^, it is totally unsuitable for the warm rolling process at 423 K with 10% reduction ratio. The detailed images obtained from warm rolled Al_81_Cu_13_Si_6_ alloy are shown in Supplementary Fig. S4. In contrast, the large number of cracks found on the surface of the (Al_81_Cu_13_Si_6_)_97_(Sn_57_Bi_43_)_3_ alloy after warm rolling under the same conditions as for the Al-Cu-Si bimodal eutectic alloy has been filled by the Sn-Bi rich phases. Even though some rather large cracks were not completely filled, almost all micron-scale cracks were filled, as shown in Fig. 6d–f. The threshold crack size which can be healed by the warm rolling at 423 K is approximately 28 μm. As shown in the inset images, especially, the regions filling the cracks display an anomalous eutectic structure, which suggests that the Sn-Bi anomalous eutectic region dynamically filled the cracks during warm rolling at 423 K. Moreover, flow patterns, which appear like traces of a trickling fluid, are observed in the filled crack areas (Fig. 6f). This gives strong evidence that the Sn-Bi anomalous eutectic phases are in liquid state at 423 K and they can dynamically fill the cracks occurring during the warm rolling process. The detailed images according to the filled cracks after warm rolling are shown in Supplementary Fig. S5. These findings indicate that the present Al-Cu-Si-Sn-Bi alloys with liquid phase separation can be applied as self-healing and/or crack filling materials, utilizing a combination of precipitation and low melting materials without the need for external healing agents.

## Discussion

XRD analysis suggests that the Sn and Bi phases formed as eutectic solid solution phases when these elements were added to the Al-Cu-Si bimodal eutectic alloy. Moreover, Sn and Bi independently form eutectic Sn-Bi droplets during solidification, as shown in the SEM images. This microstructural features can be explained by liquid phase separation and via a monotectic reaction^31–34^. The DSC traces clearly demonstrate that the Al-Cu-Si-Sn-Bi alloy system undergoes two thermal events during heating, which gives experimental evidence for liquid separation (miscibility gap) between (Al-Cu-Si) and (Sn-Bi). Due to the liquid phase separation relationship between (Al-Cu-Si) and (Sn-Bi), the bulk liquid separates into the two different liquid AlCuSi-rich L_1_ and SnBi-rich L_2_ phases at high temperature (more than 850 K). During solidification, the L_1_ phase first transforms to the bimodal eutectic structure consisting of (Al + Al_2_Cu) eutectic colonies and (Al + Al_2_Cu + Si) eutectic matrix. Therefore, the alloy has an entrapped L_2_ + bimodal eutectic structure after passing through the temperature range from 830 to 780 K. When the bimodal eutectic structure develops, the coarse eutectic colonies consisting of Al + Al_2_Cu are preferentially formed^37^. Thus, the partially solidified L_1_ (L_1_’), which forms the ultrafine eutectic matrix, contains a large amount of solutes such as Si and Sn. As solidification progresses further, a monotectic reaction occurs, where the supersaturated L_1_’ transforms (L_1_’ → Al + Al_2_Cu + Si + Sn-rich L_1_”), and finally Sn-rich L_1_” forms the small Sn particles displayed in Fig. 2e. This is the reason for the monotectic microstructure in the bimodal eutectic area^33^. Subsequently, the remaining Sn-Bi rich L_2_ produces the Sn-Bi anomalous eutectic droplets, as shown in Figs 2c and 3a. Therefore, the solidification process can be summarized as follows: the primary liquid separation, L → L_1_ (Al-Cu-Si rich) + L_2_ (Sn-Bi rich), progresses at high temperature, and the eutectic reaction as well as the monotectic reaction, L_1_ → (Al-Al_2_Cu eutectic colony) + L_1_’, L_1_ → (Al-Al_2_Cu EC) + (Al-Al_2_Cu-Si-Sn eutectic matrix) subsequently occur with decreasing temperature. At this stage, the alloy consists of the bimodal eutectic structure and entrapped Sn-Bi rich L_2_, which can be explained as L → L_1_ + L_2_ → (Al-Al_2_Cu EC) + L_1_’ + L_2_ → (Al-Al_2_Cu) + (Al-Al_2_Cu-Si) + L_1_” + L_2_ → (Al-Al_2_Cu) + (Al-Al_2_Cu-Si-Sn) + L_2_ in accordance with the decreasing temperature. Finally, the alloy forms a trimodal eutectic microstructure because the Sn-Bi-rich L_2_ phases form the Sn-Bi eutectic droplets, as shown in the SEM images of Fig. 2. Furthermore, these 2 different eutectic regions, i.e. the Al-Cu-Si bimodal eutectic and the Sn-Bi anomalous eutectic structure, have distinctly different melting/solidification behavior, which is the reason that the Al-Cu-Si-Sn-Bi composites can be applied for self-healing. From the nanoindentation results, it is noticed that the addition of Sn and Bi has scarcely influence on the hardness of the Al-rich matrix, whereas causes a notable decrease in the indentation modulus. Moreover, the indentation values such as hardness and modulus of the Sn-Bi-rich droplets is quite lower. In other words, the additional Sn and Bi-induced decrease of the modulus allude to that the Sn-Bi-containing alloy is less stiff, indicating that the alloy involving the Sn and Bi could be presumably more advantageous in a post-processing such as the rolling.

As shown in Fig. 5a–d, brighter contrast Sn-Bi-rich particles are formed on the indented surface after heat treatment at 423 K for 25 min. Moreover, the Sn-Bi-rich particles have a tendency to precipitate from the Al-Cu-Si rich matrix around the highly deformed corners of the indents (high stress/strain regions). In general, the atomic diffusion rate in the highly strained regions is higher than in stress-free regions due to the high density of dislocations and the lower energy barrier for atomic diffusion. Moreover, the interfaces between phases are well-known diffusion paths where the atoms can easily move^22,24,30^. Hence, the eutectic alloy has a strong potential for higher diffusion rates of solutes and thereby precipitation, which promotes crack filling and/or self-healing of metallic materials. Our findings indicate that the Sn and Bi solutes in the supersaturated Al-based eutectic alloy, as shown in the Fig. 3c,d, can act as efficient healing agents via segregation and/or precipitation. Furthermore, as shown in Fig. 5e and f, it has also been confirmed that the cracks located underneath the indents can be filled by the Sn-Bi-rich particles after the heat treatment. This indicates that open defects such as cracks and voids can be healed through the formation of precipitates and segregation, which fill up the open defects.

In addition, another healing process triggered by the low melting temperature metallic healing agents is possible. The 420 K which has observed from DSC analysis that the Sn-Bi anomalous eutectic phases exist in liquid state is quite lower than that of other reported Al-based alloy systems which have self-healing effects induced by metallic materials with low melting temperature^31–33^. Hence, the present alloy system has a merit in that the self-healing effect occurs at relatively lower temperatures originating from the eutectic reaction. As shown in Fig. 6, cracks that are formed upon warm rolling can be dynamically filled by the Sn-Bi anomalous eutectic healing agents during the forming operation. The anomalous eutectic microstructure of the crack filling areas (inset in Fig. 6e) and the flow patterns observed for as-rolled specimens (Fig. 6f) indicate that the Sn-Bi eutectic is in the liquid state at relatively low temperature (423 K) and flows to fill up the cracks formed during rolling. This is triggered by the external pressure imposed during rolling and the material fills up the crack on solidification.

**Figure 7:**
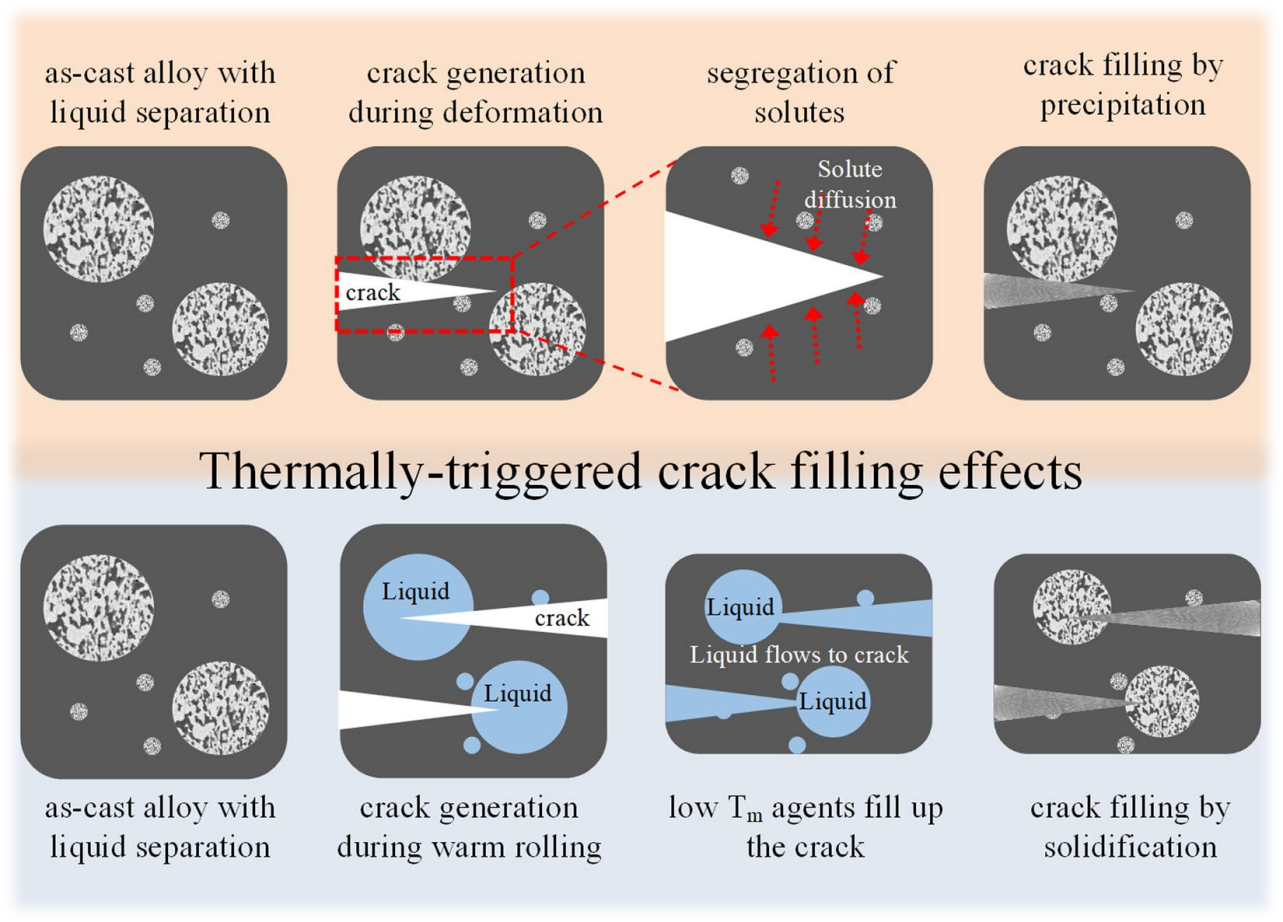
Schematic illustration of the thermally-triggered crack filling and dual self-healing effects proposed form this work.

Usually, the incorporation of alloys with low melting temperature can be tricky. Until now, some methods have been utilized by infiltrating low melting temperature alloy agents using ex-situ processes such as incorporation of hollow ceramic tubes or carbon nanotube filled healing agents, which are required as additional supply of the healing agents from the outside^20,21,23^. Such ex-situ methods face challenges such as homogeneous distribution of the healing agent container and cracks have to pass across the container. In contrast, using eutectic systems and liquid phase separation allows developing alloys with homogeneously distributed low melting healing agents. These alloys can be fabricated by simple casting processes, and the volume fraction of healing agents can be easily modified by microalloying scenario. Moreover, using low melting Sn- and Bi-based eutectic healing agents allows to exploit the self-healing phenomenon at relatively low temperatures. Furthermore, the present alloy system exhibits a dual self-healing effect including precipitation and low melting temperature, which can overcome the drawbacks facing the self-healing effects in conventional metallic materials. For precipitation, it is generally known that once the supersaturated solid solution has undergone the precipitation reaction, the solute content in the matrix region will attain the equilibrium state. This makes it almost impossible to repair the crack again. Hence, some alloys with self-healing characteristics based only on precipitation may only be feasible for filling cracks just in a single healing cycle. This concern also holds for the self-healing effect using low melting temperature agents. Therefore, the alloys suggested in this study, which exhibit a thermally-triggered dual self-healing effect via precipitation and low melting temperature agents as shown in Fig. 7, have the advantage that they can simultaneously realize both effects. Hence, this class of easily castable alloys utilizing phase separation upon solidification is valuable for the creating new metallic self-healing materials.

In the present study ultrafine multi-modal eutectic composites containing liquid phase separated anomalous eutectic phases are investigated. Due to the liquid phase separation, Sn and Bi tend to separate from Al, Cu, and Si in the liquid state, i.e., Al-Cu-Si-rich and Sn-Bi-rich liquids with different melting temperatures coexist. Consequently, the (Al_81_Cu_13_Si_6_)_100−*x*_(Sn_57_Bi_43_)_*x*_ composites consisting of an ultrafine bimodal eutectic matrix and anomalous eutectic phases as two different liquids solidify in a different way. The homogeneous distribution of the Sn-Bi anomalous eutectic phases and the existence of two different melting temperatures enables thermally-triggered dual self-healing phenomena via precipitation and the low melting point of the Sn-Bi eutectic phase. During heat treatment the Sn and Bi solutes tend to segregate near highly strained regions and fill open cracks. Moreover, the melted Sn-Bi eutectic phase dynamically fills the cracks that evolve during warm rolling at 423 K. These findings indicate that Al-Cu-Si-Sn-Bi alloys can be applied as novel and efficient metallic self-healing materials.

## Methods

The (Al_81_Cu_13_Si_6_)_100−*x*_(Sn_57_Bi_43_)_*x*_ composites with *x* = 0, 1, and 3 at.% were prepared by induction melting of aluminum (99.999% purity), copper (99.997% purity), silicon (99.999% purity), tin (99.99% purity) and bismuth (99.999% purity) in boron nitride (BN) coated graphite crucibles under ultrahigh purity Ar atmosphere (99.9999%). Cast samples were fabricated pouring the melt into a cylindrical rod-shape water cooled copper mold with 5 mm diameter and 50 mm length. Phase identification of composites was performed by x-ray diffraction (XRD; Shimadzu XRD-6100) with Cu Kα_1_ radiation (${\rm{\lambda }}$ = 1.5406 Å). Microstructure analysis of the as-cast samples was performed by scanning electron microscopy (SEM; JEOL JSM-6390) and transmission electron microscopy (TEM; Technai F20) equipped with an energy-dispersive spectrometer (EDS). TEM samples were prepared by conventional ion milling (Gatan, Model 600). The melting and solidification behavior was studied by differential scanning calorimetry (DSC; TA Instruments DSC Q20) under a flow of purified argon (99.999% purity). The thermal events in the DSC traces were monitored during continuous heating in the range of 350 to 850 K using a heating rate of 0.33 K/s. The mechanical properties were confirmed by the nanoindentation (CSM, NHT-X) with a maximum load of 100 mN at a loading rate of 200 mN/min. Cylindrical specimens for Vickers hardness tests were prepared by cutting the as-cast specimens to 4 mm height and then polishing them into two halves. After the polished samples were faced and bonded with each other, the top of the bonded specimens was used for performing Vickers diamond indentations under a load of 0.49 kN. For testing the self-healing properties upon heat treatment, the indented specimens were annealed at 423 K for 25 min in a vacuum induction furnace. In order to evaluate dynamic crack filling during warm rolling, the forming process was performed with 10% reduction ration at 423 K. Afterwards, the cracks generated by rolling were investigated by SEM.

## Electronic supplementary material

Supplementary information
