# Supplementary material for: Thermally-triggered Dual In-situ Self-healing Metallic Materials
Source: Sci Rep. 2018 Feb 1;8:2120. doi: 10.1038/s41598-018-19936-4 (PMC5794764; doi:10.1038/s41598-018-19936-4)
Supplement: Supplementary file 1 — Supplementary information [file 41598_2018_19936_MOESM1_ESM.pdf]

Supplementary Information

Thermally-triggered Dual In-situ Self-healing Metallic Materials

JeongTae Kim[1,2†], Hee Jin Kim[1†], Sung Hwan Hong[1], Hae Jin Park[1], Young Seok Kim[1], Yun Jung Hwang[1], Yeon Beom Jeong[1], Jun-Young Park[1], Jin Man Park[4], Baran Sarac[2], Wei-Min Wang[5], Jürgen Eckert[2,3] and Ki Buem Kim[1*]

[1]*Department of Nanotechnology and Advanced Materials Engineering, Sejong University, 209, Neungdong-ro, Gwangjin-gu, Seoul, 05006, Republic of Korea*

[2]*Erich Schmid Institute of Materials Science, Austrian Academy of Sciences, Jahnstraße 12, A-8700 Leoben, Austria*

[3]*Department Materials Physics, Montanuniversität Leoben, Jahnstraße 12, A-8700 Leoben, Austria*

[4]*Global Technology Center, Samsung Electronics Co., Ltd, 129, Samsung-ro, Yeongtong-gu, Suwon-si, Gyeonggi-do 443-742, Republic of Korea*

[5]*Key Laboratory for Liquid-Solid Structural Evolution and Processing of Materials, Ministry of Education, Shandong University, 17923 Jingshi Road, Jinan 250061, China*

*correspondence to kbkim@sejong.ac.kr

†these authors contributed equally to this work

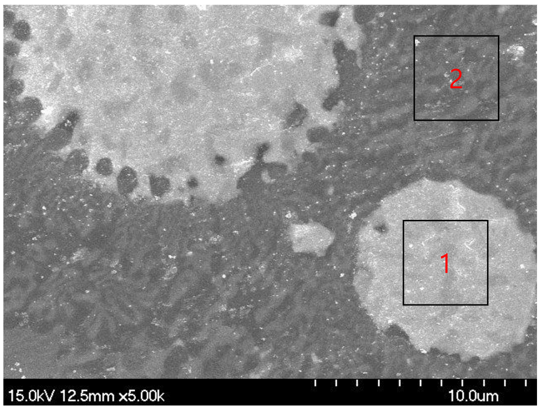

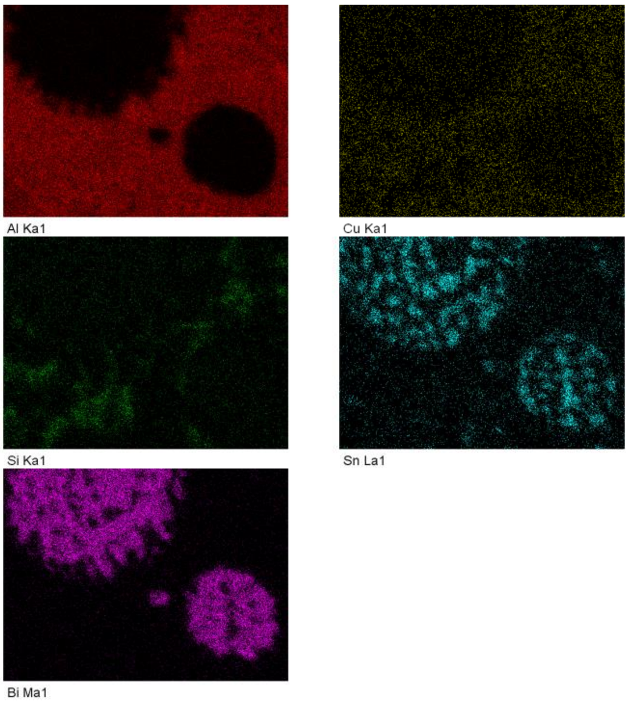

1			2		
Element	Weight%	Atomic%	Element	Weight%	Atomic%
Al K	0.53	3.19	Al K	63.01	78.16
Si K	0.06	0.35	Si K	5.23	6.24
Cu L	0.35	0.90	Cu L	27.49	14.48
Sn L	30.82	42.33	Sn L	3.59	1.01
Bi M	68.24	53.23	Bi M	0.68	0.11
Totals	100.00		Totals	100.00	

Supplementary Figure S1. SEM image and EDX analysis of as-cast $(Al_{81}Cu_{13}Si_6)_{97}(Sn_{57}Bi_{43})_3$ composite. The EDX maps clearly verify a high concentration of Sn and Bi with the spherical droplet, and beside the small amount of Sn element is observed in the matrix area. These results indicate that the Sn elements are present in the matrix as a solute.

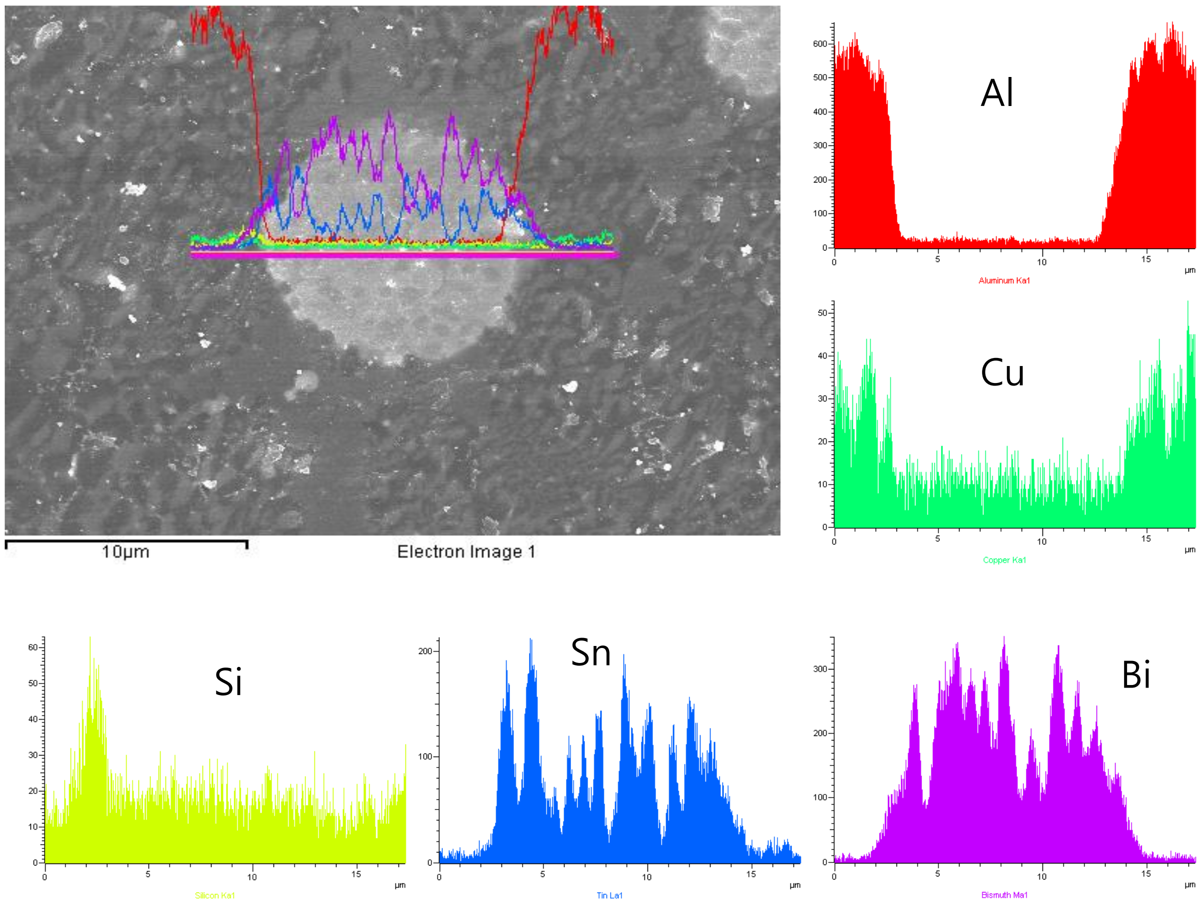

Supplementary Figure S2. SEM image and EDX line scan around the Sn-Bi-rich droplet of as-cast $(Al_{81}Cu_{13}Si_{6})_{97}(Sn_{57}Bi_{43})_{3}$ composite. The line scans show the variation of element concentration near the interface between the Sn-Bi-rich droplet and Al-rich matrix. The line scan results demonstrate the pronounced compositional difference between the droplet and matrix, as well as the concentration fluctuations indicating the alternating eutectic structure.

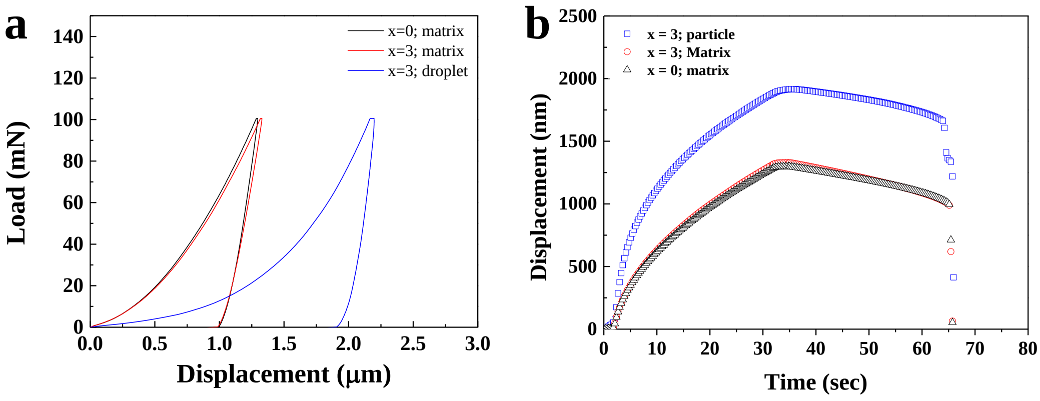

Supplementary Figure S3. (a) load-displacement (*p*-*h*) curves and (b) displacement history (*h*-*t*) curves obtained from the matrix areas of the $Al_{81}Cu_{13}Si_6$ and $(Al_{81}Cu_{13}Si_6)_{97}(Sn_{57}Bi_{43})_3$ alloys and the droplets of the $(Al_{81}Cu_{13}Si_6)_{97}(Sn_{57}Bi_{43})_3$ alloy. The trace of *p*-*h* and *h*-*t* curve and obtained from the matrix of the $Al_{81}Cu_{13}Si_6$ and $(Al_{81}Cu_{13}Si_6)_{97}(Sn_{57}Bi_{43})_3$ alloys displays the similar shape. The curve of the droplet shows the different response with the matrix area.

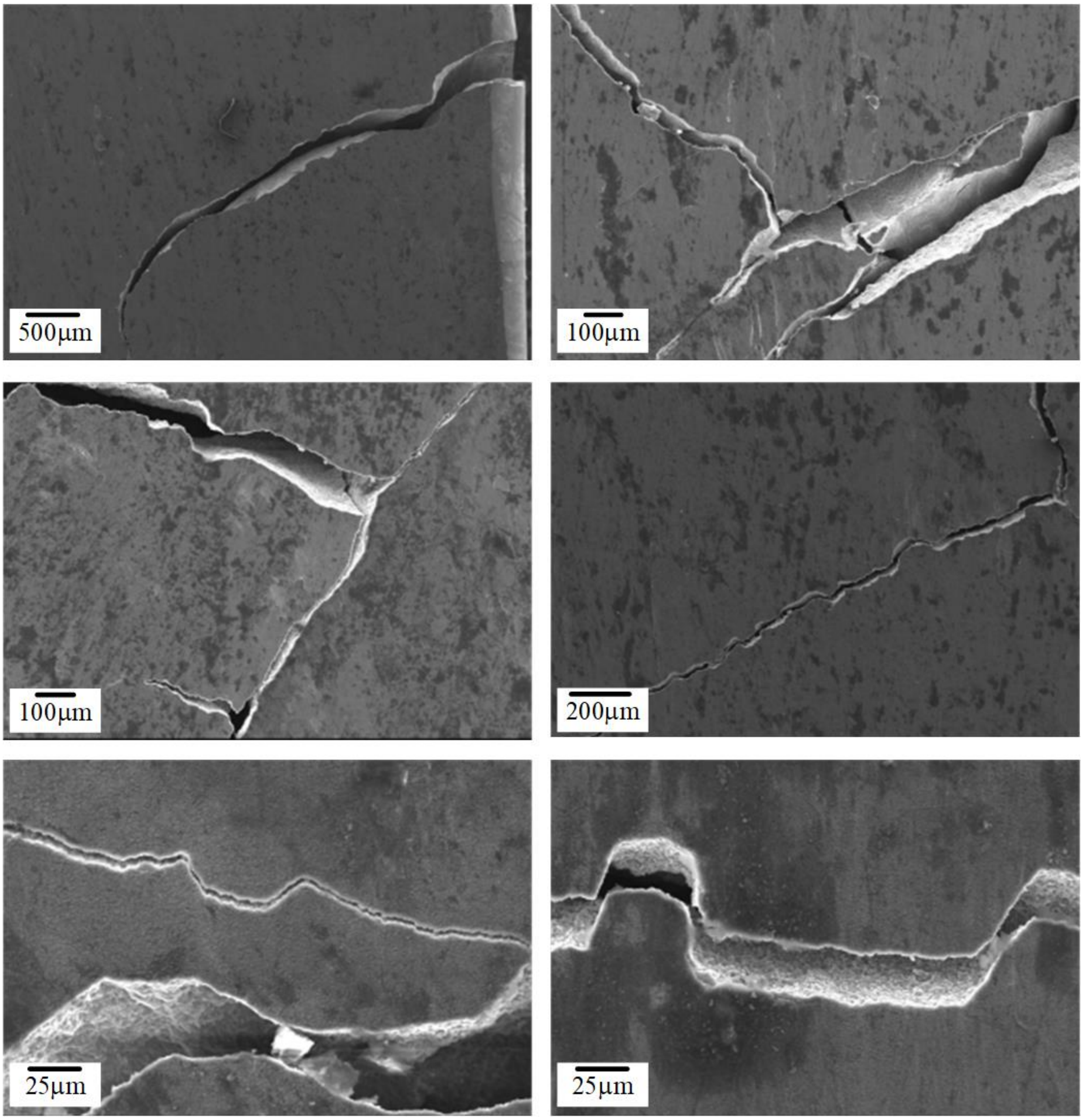

Supplementary Figure S4. SEM images after warm rolling at 423K with 10% reduction ratio for $Al_{81}Cu_{13}Si_6$ ultrafine eutectic alloy.

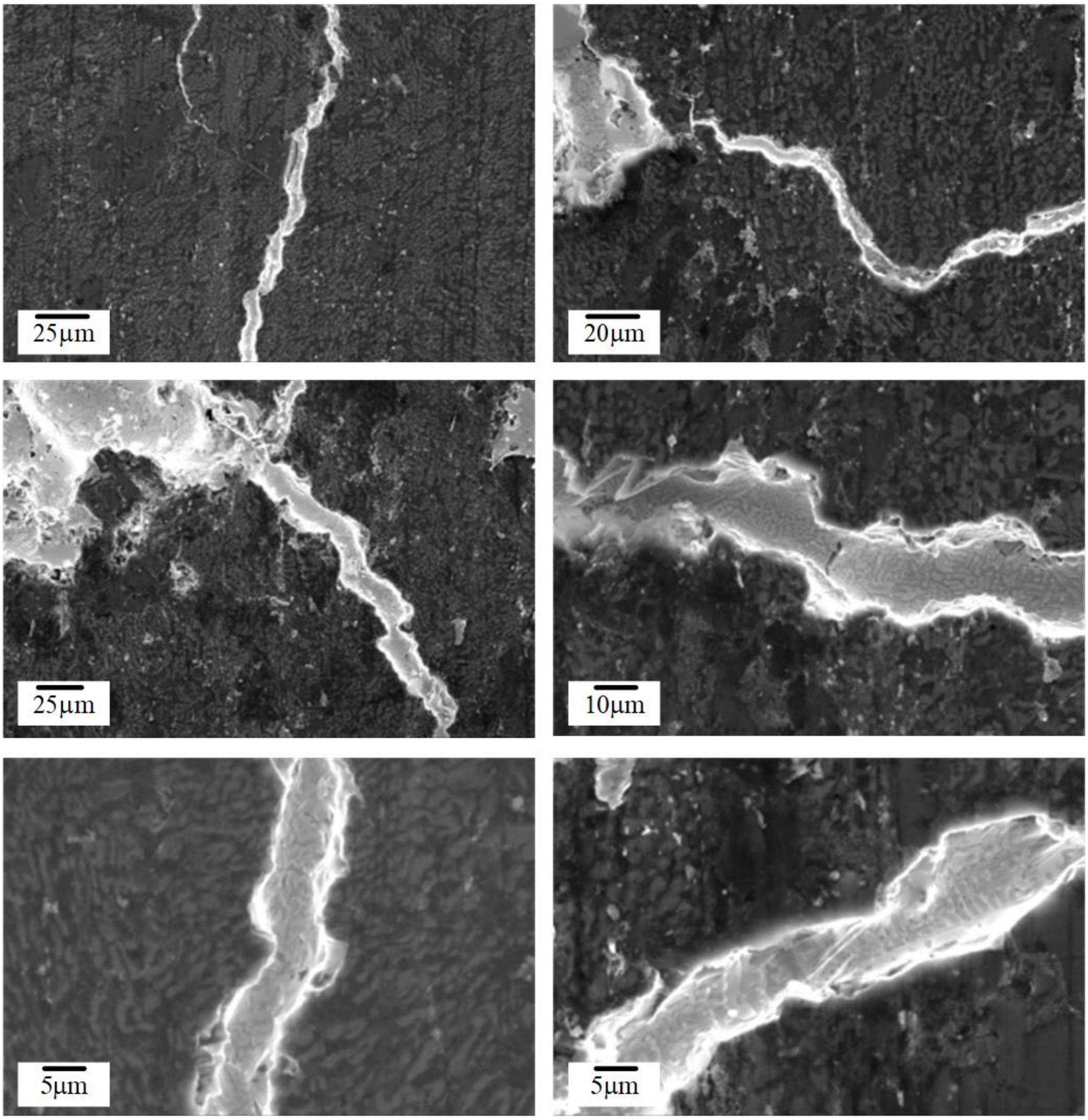

Supplementary Figure S5. SEM images of filled cracks after warm rolling at 423K with 10% reduction ratio for $(Al_{81}Cu_{13}Si_{6})_{97}(Sn_{57}Bi_{43})_{3}$ ultrafine eutectic alloy.

Phase	H_{IT} (GPa)	E_{IT} (GPa)	HV (Vickers)	Stiffness (mN/nm)	H_m (μm)
x = 0; matrix	3.12 ± 0.12	73.34 ± 1.74	289.80 ± 11.63	0.50 ± 0.02	1.30 ± 0.02
x = 3; matrix	3.14 ± 0.10	60.02 ± 2.81	291.09 ± 9.94	0.41 ± 0.02	1.33 ± 0.01
x = 3; droplet	0.95 ± 0.11	51.42 ± 3.47	87.99 ± 10.17	0.65 ± 0.01	2.20 ± 0.12

Supplementary Table S1. Nanoindentation properties of the as-cast $Al_{81}Cu_{13}Si_6$ and $(Al_{81}Cu_{13}Si_6)_{97}(Sn_{57}Bi_{43})_3$ alloys: indentation hardness H_{IT}, indentation modulus E_{IT}, vickers hadness HV, stiffness, and maximum depth H_m. It is noticed that the addition of Sn and Bi has an obscure effect on the hardness of the Al-rich matrix, whereas causes a notable decrease in the indentation modulus. Moreover, the indentation values such as hardness and modulus of the Sn-Bi-rich droplets is quite lower. In other words, the additional Sn and Bi-induced decrease of the modulus allude to that the Sn-Bi-containing alloy is less stiff, indicating that the alloy involving the Sn and Bi could be presumably more advantageous in a post-processing such as the rolling.
